# Altered protein O-GlcNAcylation in placentas from mothers with diabetes causes aberrant endocytosis in placental trophoblast cells

**DOI:** 10.1038/s41598-021-00045-8

**Published:** 2021-10-19

**Authors:** Victoria Palin, Matthew Russell, Robert Graham, John D. Aplin, Melissa Westwood

**Affiliations:** 1 Maternal and Fetal Health Research Centre, University of Manchester, Manchester Academic Health Sciences Centre, Manchester, M13 9WL UK; 2 Maternal and Fetal Health Research Centre, St Mary’s Hospital, Manchester Academic Health Sciences Centre, Manchester, UK; 3 Division of Molecular and Clinical Cancer Sciences, Stoller Biomarker Discovery Centre and Pathology Node, University of Manchester, Manchester, M13 9WL UK; 4 School of Biological Sciences, Queen’s University Belfast, Belfast, BT9 5DL UK; 5 Maternal and Fetal Health Research Centre, University of Manchester, St Mary’s Hospital, Oxford Road, Manchester, M13 9WL UK

**Keywords:** Glycomics, Glycosylation, Endocytosis, Diabetes complications, Gestational diabetes, Experimental models of disease, Reproductive disorders

## Abstract

Women with pre-existing diabetes have an increased risk of poor pregnancy outcomes, including disordered fetal growth, caused by changes to placental function. Here we investigate the possibility that the hexosamine biosynthetic pathway, which utilises cellular nutrients to regulate protein function via post-translationally modification with O-linked N-acetylglucosamine (GlcNAc), mediates the placental response to the maternal metabolic milieu. Mass spectrometry analysis revealed that the placental O-GlcNAcome is altered in women with type 1 (n = 6) or type 2 (n = 6) diabetes T2D (≥ twofold change in abundance in 162 and 165 GlcNAcylated proteins respectively compared to BMI-matched controls n = 11). Ingenuity pathway analysis indicated changes to clathrin-mediated endocytosis (CME) and CME-associated proteins, clathrin, Transferrin (TF), TF receptor and multiple Rabs, were identified as O-GlcNAcylation targets. Stimulating protein O-GlcNAcylation using glucosamine (2.5 mM) increased the rate of TF endocytosis by human placental cells (p = 0.02) and explants (p = 0.04). Differential GlcNAcylation of CME proteins suggests altered transfer of cargo by placentas of women with pre-gestational diabetes, which may contribute to alterations in fetal growth. The human placental O-GlcNAcome provides a resource to aid further investigation of molecular mechanisms governing placental nutrient sensing.

## Introduction

It is estimated that by 2030, at least 552 million people worldwide will suffer from type 1 or type 2 diabetes^1^; of these, 90 million are predicted to be women of reproductive age, increasing the incidence of pregnancy complicated by maternal diabetes. Such pregnancies are associated with adverse outcomes, including abnormal intrauterine growth, commonly leading to infants who are overgrown (macrosomic) at birth^2^ and have a greater risk of delayed neurodevelopment^3^, childhood obesity and of developing cardiovascular disease, obesity, and diabetes in adulthood^4–6^.

Fetal overgrowth is often perceived to be the consequence of the increased availability, and supply, of maternal nutrients^7,8^. This hypothesis assumes that the placenta, which forms the interface between mother and fetus, allows passive transfer of nutrients. However, both animal and clinical studies show that the placenta actively regulates transfer to the fetus^9^, modifying its structure and function to achieve nutrient transfer that is optimal for fetal development and growth. Failure of this adaptive process likely contributes to fetal growth disorders associated with maternal diabetes.

Understanding of the molecular mechanisms involved in placental cell (trophoblast) recognition of maternal nutrient availability and how this information is translated into altered function is currently limited. However, studies of other nutrient-sensing tissues have shown that nutrient flux through the hexosamine biosynthetic pathway (HBP), and the consequent modification of intracellular proteins with O-linked β-N-acetylglucosamine (O-GlcNAc), is a key regulator of cellular function^10^. Protein O-GlcNAcylation is mediated by the enzyme O-β-N-acetylglucosaminyl transferase (OGT), which uses uridine diphosphate (UDP)-GlcNAc derived from the HBP as a substrate for the addition of O-GlcNAc to serine or threonine residues of nuclear and cytosolic proteins. O-GlcNACase (OGA) is responsible for removing the amide^11^. Interestingly, rodent studies have shown high abundance of these two enzymes in placenta^12,13^.

Proteins from a wide variety of functional groups—transcription factors, signalling molecules, chaperones, cytoskeletal proteins and enzymes—are known targets for O-GlcNAcylation, hence increased HBP metabolism, which is activated by all basic units of energy (glucose, free fatty acids and amino acids)^14^, can have a profound effect on cellular behaviour. Previous studies have reported increased protein O-GlcNAcylation in blood cells isolated from patients with diabetes.

Therefore this study sought to determine whether placental proteins are differentially O-GlcNAcylated in placentas from women with type 1 and type 2 diabetes in order to identify pathways with the capacity to influence placental development/function that may be affected by the changes in HBP flux induced by the abnormal maternal metabolic milieu in pregnancies complicated by diabetes.

## Results

### Human trophoblast expresses the enzymes needed for O-GlcNAc cycling

Published data suggest that the human placenta expresses OGT and OGA^13^, though little is known about the distribution of O-GlcNAcylated proteins during gestation. Here we show that both enzymes are apparent in villous stroma and capillaries, but they localise predominantly to trophoblast, including the maternal-facing syncytiotrophoblast and the underlying progenitor cytotrophoblast both in first trimester and term placenta (Supplementary Fig. S1A–D). Protein O-GlcNAcylation was more strongly delineated in cytotrophoblasts than the syncytium in the first trimester, with particularly notable staining in cytotrophoblast nuclei and some stromal cells (Supplementary Fig. S1E,F).

### The placental O-GlcNAcome is altered in women with pre-existing diabetes

In order to understand how protein O-GlcNAcylation status is altered (specified as a difference of > twofold in abundance) in women who have type 1 or type 2 diabetes (Table 1), and whether such changes affect placental function, proteins isolated from placental lysates using sWGA pull-down were analysed by mass spectrometry. 961 proteins were identified in total; 668 and 695 in T1D and T2D placenta, respectively (Supplementary Table S1); some of which were unique to particular sample groups (Supplementary Table S2). The majority (55.7%) were located to the cytoplasm, then the nucleus (17.1%), followed by plasma membranes proteins (Supplementary Fig. S2) and included are known OGT targets such as actin, tubulin, a subset of ribosomal proteins; OGT itself, as previously reported, was also GlcNAcylated^11,15,16^. Although not present in all biosamples, GFAT, known as the rate-limiting enzyme in the HBP^17^ was identified in the T2D GlcNAcome. This step is referred to as the rate-limiting step in the HBP, as GFAT function is subject to feedback and partial inhibition through the accumulation of UDP-GlcNAc.

**Table 1:** Demographics, obstetrics and biophysical data for patient participants with type 1 diabetes (T1D) and type 2 diabetes (T2D) and BMI-matched controls used for mass spectrometry.

	T1CON (n = 5)	T1D (n = 6)	T2CON (n = 6)	T2D (n = 6)	p
Maternal age	36 (33–39)	32.5 (19–37)	33 (27–42)	36 (26–42)	(a) ns (b) ns
Maternal BMI (kg/m^2^)	22.11 (21.9–24)	23.8 (20–33.8)	31.0 (30.1–32)	36.5 (31–41)	(a) ns (b) ns
Smoker (%)	0	0	0	0	(a) ns (b) ns
Ethnicity (% Caucasian)	60.00	83.33	50.00	83.33	(a) ns (b) ns
Gestation (weeks)	39.1 (36.7–41.3)	37.5 (35.6–39.0)	39.0 (38.4–41.0)	38.1 (36.0–39.0)	(a) ns (b) *
Fetal sex (Males; %)	60.00	83.33	50.00	83.33	(a) ns (b) ns
Birth weight (g)	3080 (2920–3800)	3682 (2660–4340)	3590 (3180–4200)	3360 (2990–3867)	(a) ns (b) ns
Individualised birthweight centile	49.5 (13.8–80)	99.5 (78–100)	55.8 (34–90)	67 (35–95)	(a) ns (b) ns

Data are median (range). P-values were calculated for (a) T1D compared to controls with a BMI 20–25 (b) T2D compared to controls with a BMI 30–35, using Mann–Whitney test, or Fishers exact test for smoking, ethnicity and fetal sex only.

*p = 0.05.

**Figure 1:**
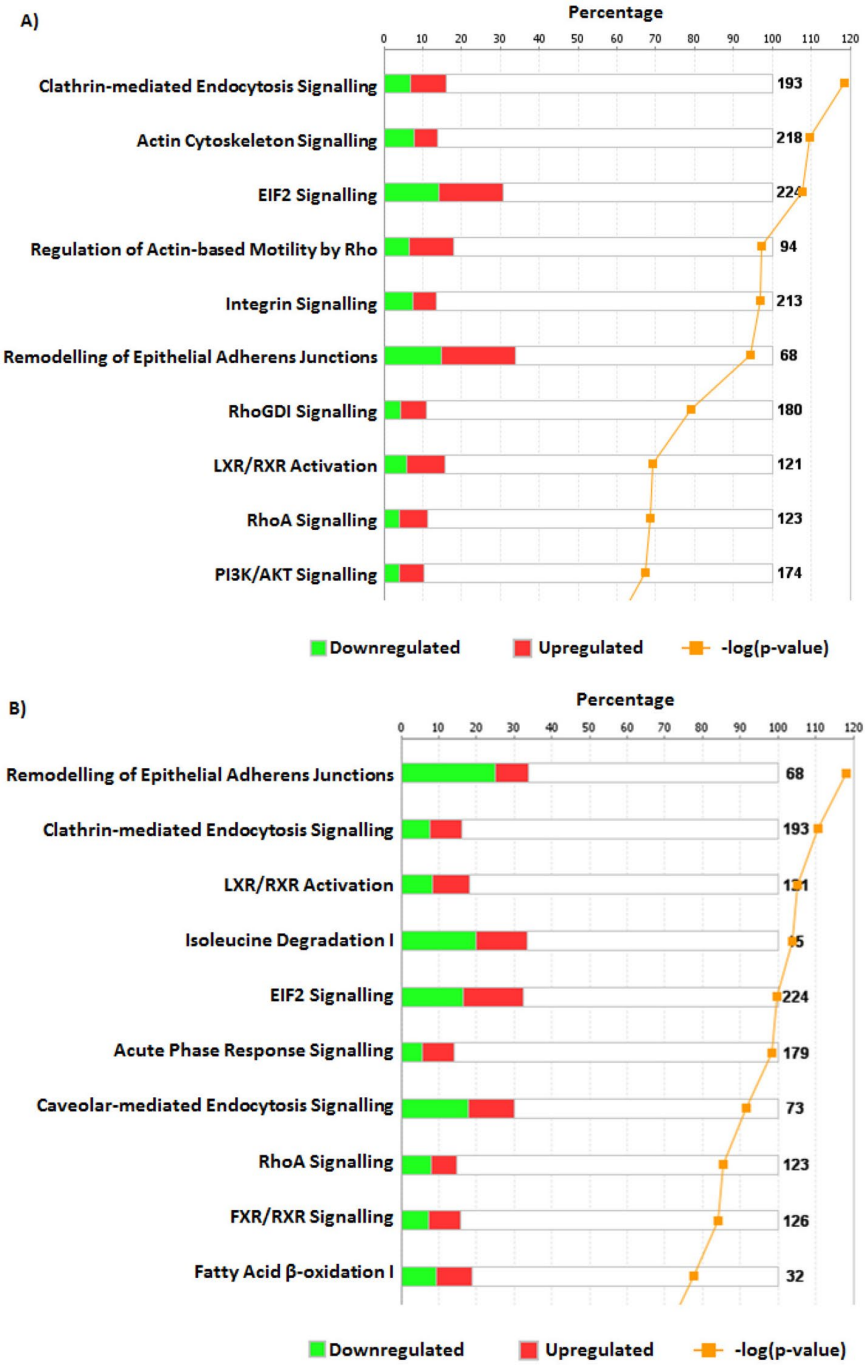
The top ten canonical pathways identified using ingenuity pathway analysis (IPA) of placental O-GlcNAcylated proteins with a > twofold change in abundance between control and maternal disease. Fold change was calculated as a change in abundance of placental proteins from mothers with T1D compared to mothers with a normal BMI (20–25) (**A**) or mothers with T2D compared to mothers with an obese BMI (> 30) (**B**). Pathways were ranked on a combination of the ratio and p-value. Ratio was calculated by the number of proteins altered (up—red; down—green) in the dataset compared to the total number of known proteins (indicated to the right hand side) within the canonical pathway. The p-value (orange line) indicates the significance of datasets association with the canonical pathway, as calculated by a Right-sided Fishers exact test using IPA.

Of 668 proteins present in both BMI-matched control (T1CON) and T1D placentas, 162 were differentially O-GlcNAcylated. Pathway analysis suggested that ‘clathrin mediated endocytosis signalling’, ‘actin cytoskeleton signalling’, and ‘EIF2 signalling’ are the three pathways most likely to be affected by O-GlcNAcylation (Fig. 1A). When the O-GlcNAcome of placentas from mothers with T2D was compared to that of women with similar BMI (> 30), thus controlling for changes due to obesity (T2CON), there were 696 proteins in common, 165 of which differed in abundance between the two groups. IPA predicted impacts on ‘remodelling of epithelial adherens junctions’, ‘clathrin-mediated endocytosis signalling’ and ‘LXR/RXR activation’ (Fig. 1B).

**Figure 2:**
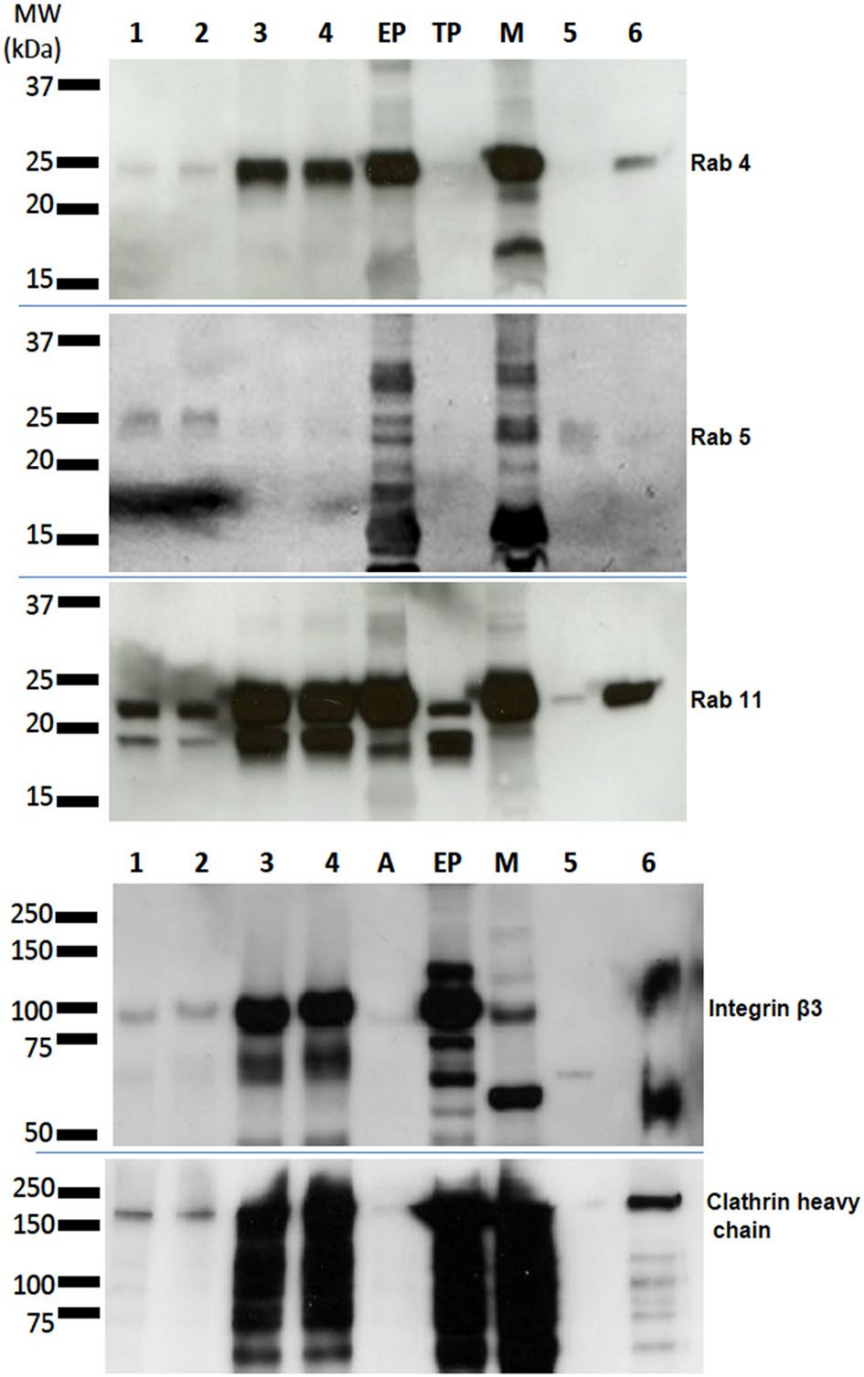
Components of clathrin-mediated endocytosis are O-GlcNAc modified. Western blot analysis of O-GlcNAc-modified proteins isolated by sWGA-lectin pulldown from term placenta lysates (n = 6; pooled) obtained from mothers with T2D (1), or BMI-matched control (2). The remaining supernatants, depleted of O-GlcNAc proteins (lanes 3 and 4, respectively). (A) Plain, unconjugated agarose beads exposed to tissue lysate and precipitate were used as a negative control, loaded to show any nonspecific binding. Three positive controls (40 μg each): (EP) first trimester human placenta (TP) term human placenta and (M) mouse brain were loaded to demonstrate the specificity of the primary antibodies. Lane (5) BeWo lysate, from control untreated cells, following sWGA-lectin enrichment and (6) depleted BeWo supernatant. Membranes were probed with antibodies specific for RAB4, RAB5, RAB11, integrin β3 and clathrin heavy chain. Full blots are shown in Supplementary Fig. S3.

As clathrin-mediated endocytosis (CME) was amongst the top three canonical pathways affected by changes to protein O-GlcNAcylation for both T1D and T2D, and endocytosis is a major function of the placenta and therefore changes to CME as a result of altered O-GlcNAcylation may have implications for fetal health, we chose to validate predictions arising from our characterisation of the placental O-GlcNAcome by selecting this pathway for further analysis. 31 proteins identified in T1D or T2D O-GlcNAcomes were related to CME, representing 16% of the proteins known to participate in this pathway; 26 were present in both datasets (83.9% crossover) and 5 were unique to one of the two O-GlcNAcomes (Supplementary Table S3). Western blotting of enriched lysates was used to test a sub-set of CME-related proteins (clathrin heavy chain, Rab 4, 5 and 11, and integrin beta-3) and showed in each case that a small fraction of the total protein was O-GlcNAcylated (Fig. 2; Supplementary Fig. S3). Peptide mass analysis revealed that multiple serine and threonine residues in key CME pathway proteins are differentially dehydrated or HexNAc modified in T1D and T2D placentas compared to their respective control groups (Table 2).

**Table 2:**
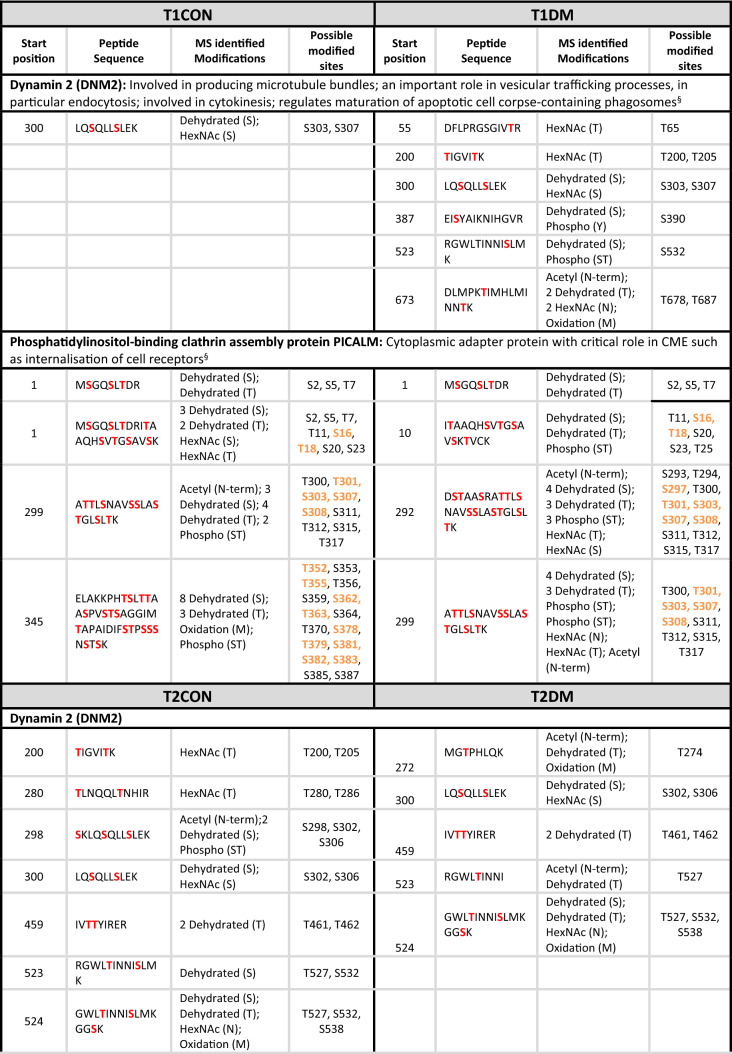

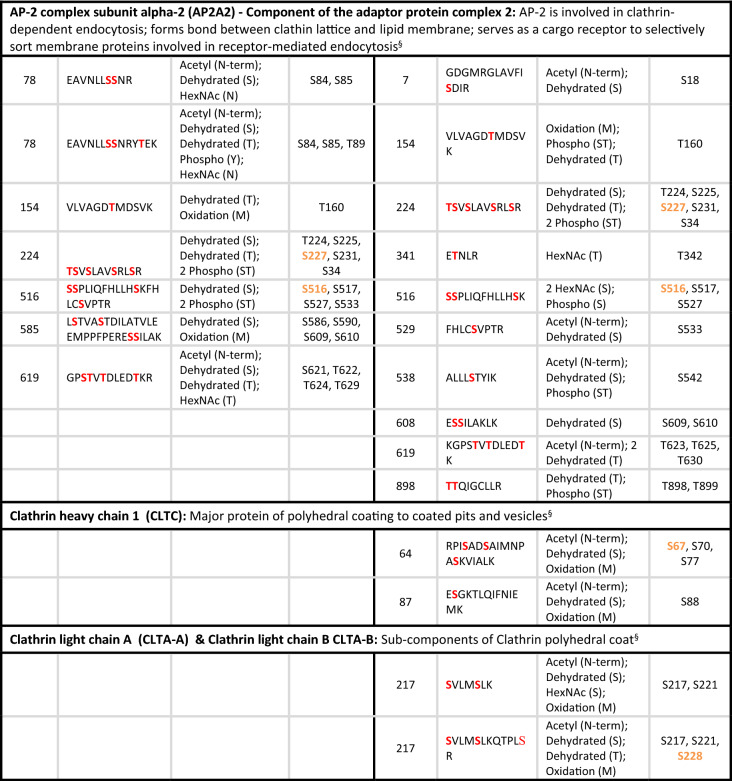
Peptide sequences for the clathrin mediated endocytosis proteins identified in the placental GlcNAcomes: showing possible residue modification in placentas compared to BMI-matched controls.

Sites shown in red were identified as modified by the mass spectrometry analysis of term placenta. Sites shown in orange were also predicted as O-GlcNAc-modified sites by the online prediction tool: available here http://www.cbs.dtu.dk/services/YinOYang/ accessed January 2021.

^§^Uniprot.org.

In both the T1DM and T2DM, the clathrin heavy chain is less O-GlcNAcylated compared to the respective control, but although previous mass spectroscopy studies identified clathrin heavy chain as O-GlcNAc modified^18^ the finding was never validated and it is not known how modification at the 4 candidate residues identified (Table 2) affects function. Similarly, the adapter protein, AP2A2 was less represented, i.e., less O-GlcNAc-modified in the enriched placental lysates from mothers with T2D, and two of the predicted O-GlcNAc sites (S227 and S516) were identified as O-GlcNAc-modified by the current study (Table 2). In contrast, the adaptor protein PICALM was more O-GlcNAcylated in the enriched placental lysates from mothers with T1D, where 6 of the sites identified in the MS analysis matched those predicted by an online algorithm (Table 2). Dynamin 2, which is involved in vesicle scission during endocytosis, was also more O-GlcNAcylated in placentas from women with both types of diabetes and we identified numerous modification sites withing the protein.

Together these data suggest that proteins involved in the formation of clathrin-coated pits, endosomal traffic and the intracellular processing of endocytosed cargo are differentially O-GlcNAcylated in placentas from mothers with diabetes compared to those from controls. However currently it is not known how altering the O-GlcNAcylation status of these proteins affects their individual function or the cumulative effect on the CME pathway. Therefore, we next sought to determine the effect of activating the HBP on the uptake of a classical CME cargo, the transferrin receptor (TfR), which is well characterised in syncytiotrophoblast^19^. Furthermore, although not differentially O-GlcNAc-modified in diabetes compared to the BMI-matched controls, the TfR was identified in all placental O-GlcNAcomes, with 5 modified residues (Table 3), and its ligand, transferrin, was more O-GlcNAcyalted (19.5-fold) in placentas from women with T2D (Supplementary Table S3).

**Table 3:**
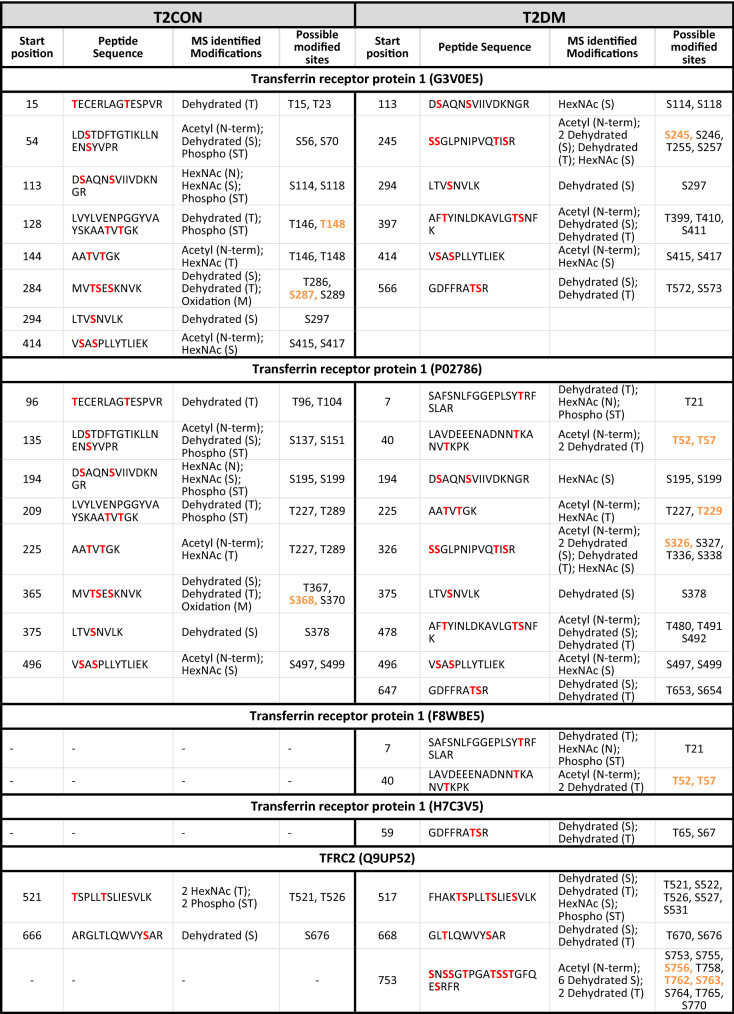
Peptide sequences for the transferrin receptor identified in the placental GlcNAcomes: showing possible residue modification in T2D placentas compared to obese-matched controls (T2CON).

Sites shown in red were identified as modified by the mass spectrometry analysis of term placenta. Sites shown in orange were also predicted as O-GlcNAc-modified sites by the online prediction tool: available here http://www.cbs.dtu.dk/services/YinOYang/ accessed January 2021.

^§^Uniprot.org.

### Altering protein O-GlcNAcylation affects clathrin-mediated endocytosis in placental cells

**Figure 3:**
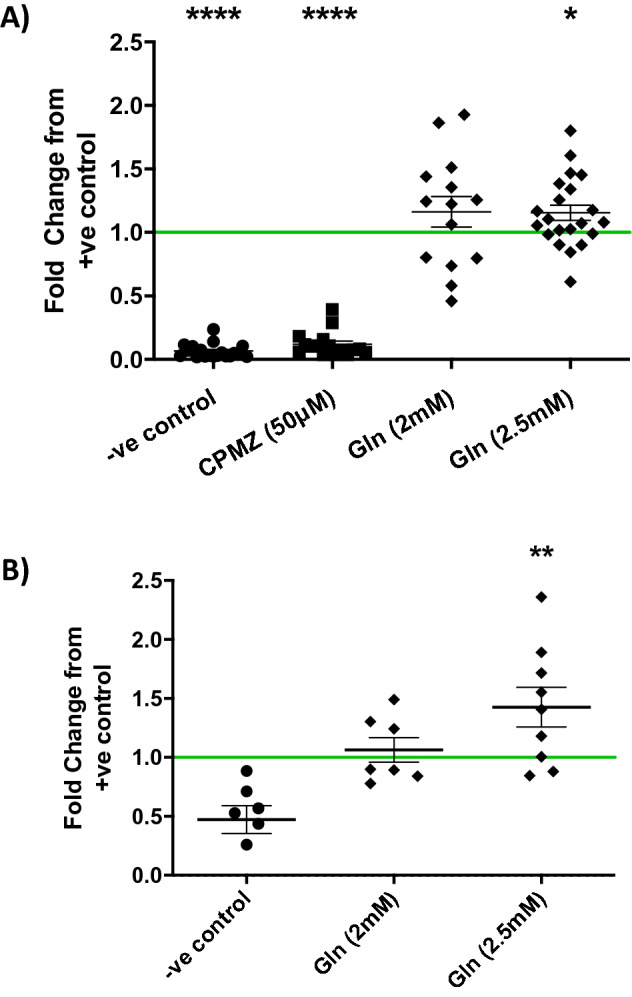
**(A) **Flow cytometric analysis of clathrin-mediated endocytosis of fluorescently labelled transferrin (Alexa 488) in BeWo cells. Cells were pre-cultured (48 h) with 2 mM (n = 14) or 2.5 mM (n = 21) glucosamine (Gln), before the addition of transferrin (6.25ug/ml) for 15 min at 37 °C (± Chlorpromazine, CPMZ clathrin inhibitor). Internalised transferrin is displayed as mean fluorescence per event in 10,000 events, as a fold change from the positive control (indicated by the green intercepting line). (**B**) Assessment of clathrin-mediated endocytosis of fluorescently labelled transferrin in first trimester human placental tissue (8.5–12 weeks gestation). Tissue explants cultured (48 h) with glucosamine ex vivo were further cultures in serum depleted medium with glucosamine (3 h) to measure the rate of uptake of transferrin (50 μg/ml). Following culture tissues were acid washed (20 s) and lysed (RIPA buffer). Fluorescent intensity of lysate read on a plate reader and normalised to total protein concentration. −ve: negative control tissue was not exposed to transferrin. Data displayed as mean and SEM, Wilcoxon signed ranked statistical analysis was used, where *p = 0.05, **p = 0.01, ***p =  < 0.001 and ****p =  < 0.0001 versus positive control.

Initial experiments demonstrated that, as in other cells and tissues^20,21^, treatment of the human placental cell line BeWo, with glucosamine causes an increase in global protein O-GlcNAcylation (Supplementary Fig. S4). Next, BeWo cell monolayers that had been exposed to fluorescently-labelled transferrin were dissociated and analysed by flow cytometry; Fig. 3A shows that internalisation and accumulation was increased 1.16-fold (p = 0.02) in cells exposed to 2.5 mM glucosamine (48 h). Women with pre-gestational diabetes may have an altered metabolic milieu from the very beginning of pregnancy, when the placental blueprint, which is key to pregnancy success, is established. Also, CME is particularly important in first trimester placenta as at this gestation, the syncytiotrophoblast does not express the caveolins required for other forms of endocytosis^22,23^. Therefore, tissue explants from first trimester human placenta were chosen as a physiological model for confirming our findings in BeWo. Global O-GlcNAcylation was enhanced by treatment of these explants with glucosamine (Supplementary Fig. S4) and uptake of transferrin was also increased (Fig. 3B; p = 0.01).

## Discussion

This work documents the O-GlcNAcome of human placenta, reporting > 750 predominantly nucleocytoplasmic O-GlcNAcylated proteins. Immunolocalised O-GlcNAcylation sites are most abundant in trophoblast, the exchange epithelium of the placenta, and the fetal capillaries within villous tissue, suggesting roles in regulating trophoblast and endothelial function. We quantified alterations to global protein O-GlcNAcylation in placentas exposed to an altered metabolic environment in vivo—pre-existing maternal diabetes—to investigate whether this molecular switch might contribute to an aberrant phenotype. Comparisons were made with overtly normal placentas from obese women, thus controlling for elevated BMI in the T2D group. Pathway analysis was implemented to help formulate hypotheses regarding the role of GlcNAcylation in tissue function, focussing on villous syncytiotrophoblast as the maternally-facing cell layer.

The women recruited to our study had intensive monitoring and glucose-lowering treatment throughout their pregnancy in order to improve blood glucose control and normalise infant birthweight, but we present evidence that even in such circumstances, maternal diabetes still influences the placental O-GlcNAcome. This is a clinically relevant observation, since improved glucose control has not so far led to a suppression of overgrowth or growth restriction in pregnancies among women with diabetes^24^. A future analysis of placentas from mothers with gestational diabetes, which develops in the second trimester after the placenta is established, would provide a useful comparison. Though our findings are based on a relatively small sample size, commonalities in the pathways identified in placentas from pregnancies complicated by either type of pre-pregnancy diabetes provide some reassurance of pathophysiological relevance. OGT preference for substrate is influenced by the levels of UDP-GlcNAc^25^, so activation of the HBP can shift the profile of proteins that are O-GlcNAcylated, and therefore it was not surprising to find that some were less represented in the samples from mothers with diabetes. In addition, the modification of some residues will have been influenced by drivers of phosphorylation, and the outcome of OGT-kinase competition^26^.

Since pathway prediction is based on data archived from many cell types and tissues, it was important to identify functions of specific relevance to this tissue and the disease phenotype. O-GlcNAc regulation of translation^27^ and the actin cytoskeleton^28^ are of undoubted interest in relation to placental function. Reduced translation, as a consequence of increased eIF2α phosphorylation, reportedly contributes to placental dysfunction in pregnancies complicated by fetal growth restricted^29^, trophoblast turnover, fusion and motility are all influenced by cytoskeletal dynamics^30–33^, as is integrity of the endothelial barrier^34^. However, here we chose clathrin-mediated endocytosis (CME) for further study. This system is crucial for micronutrient transport^35^ and growth factor signalling^23^ in syncytiotrophoblast, and was a high confidence hit when comparing placenta from BMI-matched mothers with tissue from T1D or T2D. We used a pull-down-Western approach to confirm that clathrin subunits and RAB proteins, all important components of the canonical endocytic machinery, are GlcNAcylated. Numerous other CME proteins are present in the respective placental GlcNAcomes. Integrin signalling was also a high probability hit; in light of evidence that integrin beta-3 is a component of the apical syncytiotrophoblast membrane^36^ that can modulate CME^37^, we confirmed that beta-3 is O-GlcNAcylated in placenta, used the published approach^18^ of immunoblotting isolated modified proteins for individual candidates. These experiments supported our novel finding that key CME pathway components—clathrin, RAB5 and RAB11- are OGT targets, though no quantitative readout regarding a change in O-GlcNAcylation status between normal pregnancy and diabetes could be made due to the limited sensitivity of this method for detecting subtle changes in the level of O-GlcNAcylation, especially when multiple sites, each of which could be more or less O-GlcNAcylated, are present. Nonetheless, the date indicate that signalling through the HBP might alter the uptake of factors central to placental function and fetal wellbeing.

Although endocytosis can be achieved by several different mechanisms^38^, CME is particularly important in early placenta, as syncytiotrophoblast at this time lacks the caveolins required for the other major endocytic pathway^22,39^. The clathrin heavy chain (CLTC) has two isoforms, CHC17 and CHC22, with 85% homology^40^; both were identified in the placental O-GlcNAc-ome and were less O-GlcNAc-modified in T2D compared to the obese control. Little is known about placental CHC22 but when induced in mouse skeletal muscle it has a distinctive role in the formation and expansion of GLUT4 storage vesicle^41^, which in placenta may lead to abberant uptake and transfer of glucose to the fetus.

Typically, an increase in endocytosis represents a mechanism for reducing transporter expression at the cell membrane^42^; however, if increased internalisation were coupled with enhanced transporter recycling to the cell membrane, nutrient uptake could be amplified. O-GlcNAcylation of proteins can influence subcellular localisation, and it will be important to examine its effect on presentation of specific nutrient transporters at the maternal-facing surface^43^.

We chose a well-characterised transporter, the transferrin receptor (TfR), also known to be present at the apical syncytiotrophoblast membrane, where it mediates placental iron uptake^44^. Evidence for GlcNAcylation was obtained by mass spectrometry, with **T**21 in the cytoplasmic tail region motif responsible for activating endocytosis^45^, dehydrated in T2D,and **T**52 and **T**57, situated in close proximity to the ‘stop-transfer sequence’ (residues 58–61)^40^, dehydrated in T2D but not the obese control, suggesting O-GlcNAc may regulate the stop-transfer-sequence, altering transferrin uptake. Finally, two dehydration sites within the ectodomain, **T**653 and **S**654, are close to a highly conserved RGD motif (646–648) crucial for transferrin binding to the TfR. However, the current paucity of information about which residues are key to activity means that further experiments will be required to establish the functional consequences of changes in O-GlcNAcylation.

TfR expression increases in placentas of mothers with diabetes^46^, which may also allow for a greater accumulation of iron, though data on the deposition of iron in such placentas are currently lacking. In a rat model of diabetes, iron supplementation led to increased deposits of iron in pancreas and heart, which correlated with increased production of reactive oxygen species^47^.

In non-pregnant individuals, diabetes is often associated with increased serum ferritin levels^48^, therefore if mothers with diabetes have high ferritin levels, it is possible that altered O-GlcNAcylation of CME proteins leading to increased CME of transferrin could result in increased placental accumulation of iron. Such iron may increase the production of free radicals and oxidative stress in the placenta, increasing tissue damage; indeed, fibrin deposits, a marker of placental damage, are a common morphological finding in placentas of mothers with diabetes^49^.

Although the majority of fetal growth occurs after the first trimester, the rate of human placental growth^50^ and nutrient transport^51^ is fastest during the first and early second trimesters. Moreover, there is now a wealth of evidence from both clinical^52^ and animal^53^ studies to suggest that whilst the clinical consequences of many pregnancy complications, including fetal growth disorders, are not apparent until the third trimester, their origin lies in abnormal placental growth and function during early development. Hence our finding that CME within first trimester placental explants is altered by HBP activation has pathophysiological relevance as, unlike in gestational diabetes, mothers with pre-existing diabetes may have an abnormal metabolic milieu from the very beginning of pregnancy.

In summary, our results suggest that responses to maternal environmental cues, including those from nutrients, hormones and growth factors^38^ could be mediated by modulation of GlcNAcylation in endocytic pathways that ultimately underpin fetal growth. Trafficking of nutrient transporters such as GLUT 1&4 (glucose) and SNAT2 (system A-type amino acids) to and from the cell surface provides syncytiotrophoblast with the ability to respond rapidly to altered maternal nutrient availability^54^ and studies of other tissues have shown that CME is a key regulator of both GLUT-1 and GLUT-4^42^. Understanding the consequences of O-GlcNAcylation of the CME machinery for endocytosis of a range of ligands will contribute to a mechanistic understanding and the development of intervention strategies for fetal growth disorders typically associated with diabetes in pregnancy. Furthermore, our findings provide new lines of enquiry for gaining insight into the extracellular control of fundamental cell biology processes that might be of relevance to the pathophysiology of other tissues and systems affected by diabetes.

## Materials and methods

### Preparation of placental tissues for mass spectrometry

Tissue was obtained following delivery of a singleton infant between 37 to 42 weeks gestation (Table 1). Samples (≈1 cm3) randomly taken from the centre, middle and edge of each placenta were homogenised and analysed for protein content. Equal proportions of placental proteins (40 µg/placenta) were pooled to create four sample groups. Samples were pre-cleared using plain agarose beads (rotating 1 h at 4 °C) to remove non-specifically bound protein, then incubated with beads conjugated with succinylated wheat germ agglutinin (sWGA; Sigma UK), which recognises GlcNAc, rotating overnight at 4 °C. Subsequently, samples were centrifuged (1000×*g* for 1 min), supernatants discarded and bead pellets were thoroughly washed in ice-cold RIPA buffer before addition of standard Laemmli loading buffer and boiling at 95 °C to elute protein. Enriched GlcNAc-modified proteins were resolved by SDS-PAGE electrophoresis (7.5% acrylamide).

Proteins within the gel were stained using Coomassie blue R-25 (16.6 g/L, Bio Rad) for 2 h at RT followed by clearing in 10% acetic acid, 15% methanol. Sample lanes were cubed (approximately 2 mm^3^) then stain removed with alternating washes using ammonium bicarbonate and ammonium bicarbonate plus acetonitrile. Next, proteins were reduced in dithiothreitol (10 mM; 30 min at 50 °C), then alkylated using iodoacetamide (C_2_H_4_INO, 55 mM; 20 min at RT, protected from light). Proteins were digested with trypsin (Promega; UK, 1.5 ng/µL; overnight at 37 °C) and peptide fragments eluted from the gel and lyophilised in a vacuum centrifuge. Peptides were reconstituted in 0.1% formic acid and stored at − 80 °C until used.

### Mass spectrometry

Peptides were analysed on a 5600 TripleTOF mass spectrometer (AB Sciex) and NanoAcquity High Performance Liquid Chromatography (HPLC; Waters). Samples were separated on a C18 analytical column (Waters BEH 75 μm × 25 cm) on a linear gradient from 3 to 40% formic acid (0.1%) in HPLC grade acetonitrile over 40 min at a flow rate of 300 nL/min.

Spectral data were extracted from the raw data to a mascot generic format file with the instrument vendors’ AB_SCIEX_MS_Converter program. Data were searched with the MASCOT engine against SwissProt and species restriction set to *H. sapiens*. Mascot also searches for fixed and variable modifications. The variable search parameters were set to include: phosphorylated forms of serine, threonine or tyrosine amino acids, as well as dehydrated serine or threonine residues, which would indicate the loss of a modification at the respective site (Supplementary Table S4). Variable modifications also searched for N-acetylhexosamine (HexNAc) of serine, threonine or asparagine residues, for a monoisotopic mass of 203.0794. This parameter was used to identify peptides and, therefore, parent proteins with the addition of O-GlcNAc, should the modification survive the collision-induced dissociation gas phase; dehydrated residues indicated a loss of phosphate or GlcNAc.

DAT files from Mascot were analysed using Scaffold (Proteome Software), quantifying proteins according to the normalised spectral abundance factor (NSAF). NSAF is defined as a ratio of the number of spectra identifying a protein divided by the protein length expressed as the number of amino acids. The NSAF score for each identified protein was then used to calculate a fold change in protein abundance in the enriched fraction, henceforth referred to as protein that is more- or less- O-GlcNAc-modified. This allowed proteins that differed by > 2 or < 0.5 fold between the sample groups to be analysed using QIAGEN’s Ingenuity Pathway Analysis (IPA) software (Ingenuity Systems, QIAGEN Redwood City, CA, USA www.qiagen.com/ingenuity).

### Western blotting

Samples enriched for GlcNAc-modified protein were resolved by SDS-PAGE, transferred to nitrocellulose membranes then probed with primary antibody (mouse anti-Rab 4, (1:1000), anti-Rab 5 (1:500), anti-Rab 11 (1:1000), anti-clathrin heavy chain (1:1000), all BD Transduction laboratories or rabbit anti-integrin β3 (1:500, Cell Signaling) overnight at 4C and visualised with horseradish peroxidase (HRP)-conjugated anti-rabbit or anti-mouse IgG secondary antibody (1 h, room temperature) and enhanced chemiluminescence. The effect of glucosamine protein O-GlcNAcylation was assessed by western blotting of cell / tissue lysates with a mouse anti-O-GlcNAc antibody (1:750, Sigma) followed by an anti-mouse Li-Cor secondary antibody (1 h at RT; 1:10,000, Li-Cor Biosciences, Lincoln; USA).

### Transferrin uptake

CME of transferrin was measured in placental cells (the human choriocarcinoma-derived trophoblast cell line, BeWo; Supplementary Fig. S5A,B) and human first trimester placental explants (Supplementary Fig. S5C). BeWo cells were cultured at 37 °C, 5% CO_2_ in medium (1:1 DMEM:Ham’s F12; Sigma) supplemented with 10% fetal bovine serum (Sigma), 2 mM l-glutamine, 100 µg/mL streptomycin, 100 IU/mL penicillin (Sigma). To simulate nutrient flux through the HBP, 2 mM or 2.5 mM glucosamine (Sigma) Glucosamine is a well well-established tool for activating the HBP^55^, as unlike glucose it is not metabolised by other pathways, enabling the HBP to be studied in isolation. Glucosamine enters the HBP downstream of the rate-limiting enzyme GFAT^56^, which is normally inhibited by raised UDP-GlcNAc levels. Cells were washed and serum-free medium containing glucosamine or chlorpromazine (CPMZ; 50 µM), an inhibitor of clathrin-mediated endocytosis, added for 1 h at 37 °C, before placing on ice for 5 min to halt all cellular trafficking. Transferrin (conjugated with Alexa-488; Thermo Fisher) was added at an optimised concentration of 6.25 μg/ml and cells incubated for 15 min at 37° before being placed on ice and washed (0.2 M acetic acid, 0.5 M sodium chloride; pH 2.8; 5 min) to remove membrane-bound transferrin. Cells were detached, washed and fixed (4% PFA, 10 min at RT), before re-suspending in 5 mM EDTA for analysis using the Accuri C6 Flow Cytometer (10,000 cells per sample). Data were analysed using the Mann–Whitney test in GraphPad Prism software (version 7), California; USA). Transferrin uptake by human placental explants was analysed by fluorescence microscopy of OCT-fixed tissue fragments (3mm^3^) following incubation with labelled transferrin, with and without 50 µM CPMZ, for 30 min, 37° and quantified in lysates of transferrin-treated tissue, pre-incubated with or without glucosamine for 48 h, on a fluorescence plate reader.

### Ethical approval and informed consent

The collection of human first trimester and term placentas was approved by the National Research Ethics Service (NRES) Committee North West—Haydock (study approval numbers 13/NW/0205 and 08/H1010/55 + 5, respectively) and in accordance with relevant guidelines and regulations. Informed, written maternal consent was obtained prior to the collection of samples.

## Supplementary Information

Supplementary Information 1.

Supplementary Information 2.
