# Supplementary material for: Altered protein O-GlcNAcylation in placentas from mothers with diabetes causes aberrant endocytosis in placental trophoblast cells
Source: Sci Rep. 2021 Oct 19;11:20705. doi: 10.1038/s41598-021-00045-8 (PMC8526670; doi:10.1038/s41598-021-00045-8)
Supplement: Supplementary file 1 — Supplementary Information 1. [file 41598_2021_45_MOESM1_ESM.pdf]

Altered protein O-GlcNAcylation in placentas from mothers with diabetes causes aberrant endocytosis in placental trophoblast cells

Victoria Palin, Matthew Russell, Robert Graham, John D Aplin and Melissa Westwood

Supplementary methods, tables and figures:

Methods

Immunohistochemistry

First trimester placenta (8-12 weeks gestation) or placenta from an uncomplicated pregnancy at term were fixed in 4% paraformaldehyde, then embedded in paraffin wax and cut into 5µm sections. Sections were boiled in 0.01M sodium citrate buffer (pH 6.0) for antigen retrieval, and then incubated with 3% hydrogen peroxide (10 mins) followed by 5% bovine serum albumin (30 mins). Primary antibodies (rabbit anti-OGT (1:100; a kind gift of Professor G Hart), chicken anti-OGA (1:100; G Hart), or mouse anti-GlcNAc (1:100; Covance)) were applied overnight at 4°C, then sections incubated with the appropriate HRP-linked secondary antibody (1:500; Dako) for 30min followed by avidin peroxidase (5µg/ml) for 45min. Immune complexes were visualised using diaminobenzidine and counterstained with Harris's hematoxylin. Sections were observed with an Olympus BX41 microscope (Olympus, UK) and photographed using a QICAM fast 1364 digital camera (QImaging, UK).

Supplementary Table 1: Placental O-GlcNAc-modified proteins identified by mass spectrometry, expressed by the normalized spectral abundance factor (NSAF).

Please see attached excel spreadsheet.

Supplementary Table 2: Unique proteins identified by sample group. Protein abundance quantified using the normalised spectral abundance factor (NSAF). NSAF is defined as a ratio of the number of spectra identifying a protein divided by the protein length expressed as the number of amino acids.

Accession Number	Identified Proteins	Molecular Weight	T1CON	T1D	T2CON	T2D
DUS3	Dual specificity protein phosphatase 3 GN=DUSP3	20 kDa	**0.0111**	0	0	0
TBB4A	Tubulin beta-4A chain GN=TUBB4A	50 kDa	**0.0046**	0	0	0
PPOX	Protoporphyrinogen oxidase GN=PPOX	51 kDa	**0.0043**	0	0	0
H7C0C1	Uncharacterized protein (Fragment)	27 kDa	**0.0042**	0	0	0
STIP1	Stress-induced-phosphoprotein 1 GN=STIP1	63 kDa	**0.0039**	0	0	0
PTPRA	Isoform 2 of Receptor-type tyrosine-protein phosphatase alpha GN=PTPRA	90 kDa	**0.0026**	0	0	0
RRAS2	Ras-related protein R-Ras2 GN=RRAS2	23 kDa	0	**0.0161**	0	0
AP2M1	AP-2 complex subunit mu GN=AP2M1	50 kDa	0	**0.0075**	0	0
CO4A	Isoform 2 of Complement C4-A GN=C4A	188 kDa	0	0	**0.0010**	0
POTEF	POTE ankyrin domain family member F GN=POTEF	121 kDa	0	0	**0.0016**	0
AT2A2	Sarcoplasmic/endoplasmic reticulum calcium ATPase 2 GN=ATP2A2	115 kDa	0	0	**0.0016**	0
M0QX68	Pregnancy-specific beta-1-glycoprotein 3 GN=PSG3	54 kDa	0	0	**0.0018**	0
SYAC	Alanine--tRNA ligase, cytoplasmic GN=AARS	107 kDa	0	0	**0.0018**	0
PTPRF	Receptor-type tyrosine-protein phosphatase F GN=PTPRF	213 kDa	0	0	**0.0018**	0
MROH5	Maestro heat-like repeat family member 5 GN=MROH5	149 kDa	0	0	**0.0019**	0
KAPCA	cAMP-dependent protein kinase catalytic subunit alpha GN=PRKACA	41 kDa	0	0	**0.0024**	0
B9A067	MICOS complex subunit MIC60 GN=IMMT	79 kDa	0	0	**0.0024**	0
LMAN2	Vesicular integral-membrane protein VIP36 GN=LMAN2	40 kDa	0	0	**0.0024**	0
ITAM	Isoform 2 of Integrin alpha-M GN=ITGAM	127 kDa	0	0	**0.003**	0
PSB5	Proteasome subunit beta type-5 GN=PSMB5	28 kDa	0	0	**0.0032**	0
VATB2	V-type proton ATPase subunit B, brain isoform GN=ATP6V1B2	57 kDa	0	0	**0.0033**	0
SYLC	Leucine--tRNA ligase, cytoplasmic GN=LARS	134 kDa	0	0	**0.0036**	0
NEP	Neprilysin GN=MME	86 kDa	0	0	**0.0045**	0
SYVC	Valine--tRNA ligase GN=VARS	140 kDa	0	0	**0.0047**	0
MVP	Major vault protein GN=MVP	99 kDa	0	0	**0.0047**	0
IF4A1	Isoform 2 of Eukaryotic initiation factor 4A-I GN=EIF4A1	40 kDa	0	0	**0.0049**	0
J3KNQ4	Alpha-parvin GN=PARVA	47 kDa	0	0	**0.0062**	0
SYCY2	Syncytin-2 GN=ERVFRD-1	60 kDa	0	0	**0.0063**	0
J3QQM1	26S protease regulatory subunit 8 (Fragment) GN=PSMC5	29 kDa	0	0	**0.0065**	0
A6NFB4	Chorionic somatomammotropin hormone 1 GN=CSH1	29 kDa	0	0	**0.0066**	0
EFHD2	EF-hand domain-containing protein D2 GN=EFHD2	27 kDa	0	0	**0.0071**	0
MK03	Isoform 2 of Mitogen-activated protein kinase 3 GN=MAPK3	38 kDa	0	0	**0.0076**	0
RHG01	Rho GTPase-activating protein 1 GN=ARHGAP1	50 kDa	0	0	**0.0078**	0
AATM	Aspartate aminotransferase, mitochondrial GN=GOT2	48 kDa	0	0	**0.0079**	0
ADRM1	Proteasomal ubiquitin receptor ADRM1 GN=ADRM1	42 kDa	0	0	**0.0084**	0
ST1A1	Sulfotransferase 1A1 GN=SULT1A1	34 kDa	0	0	**0.0086**	0
RAP1A	Ras-related protein Rap-1A GN=RAP1A	21 kDa	0	0	**0.0092**	0
GNAI3	Guanine nucleotide-binding protein G(k) subunit alpha GN=GNAI3	41 kDa	0	0	**0.0096**	0
J3KQJ1	Sulfatase-modifying factor 2 GN=SUMF2	36 kDa	0	0	**0.0106**	0
PURB	Transcriptional activator protein Pur-beta GN=PURB	33 kDa	0	0	**0.0109**	0
HPRT	Hypoxanthine-guanine phosphoribosyltransferase GN=HPRT1	25 kDa	0	0	**0.0116**	0
C9J4W5	Eukaryotic translation initiation factor 5A-2 (Fragment) GN=EIF5A2	13 kDa	0	0	**0.0148**	0
FKBP3	Peptidyl-prolyl cis-trans isomerase FKBP3 GN=FKBP3	25 kDa	0	0	**0.0152**	0
TMED4	Isoform 2 of Transmembrane emp24 domain-containing protein 4 GN=TMED4	24 kDa	0	0	**0.0161**	0
K7EQ63	Transmembrane emp24 domain-containing protein 1 (Fragment) GN=TMED1	21 kDa	0	0	**0.0178**	0
TRA2B	Isoform 3 of Transformer-2 protein homolog beta GN=TRA2B	22 kDa	0	0	**0.0181**	0
E9PIM6	Thy-1 membrane glycoprotein (Fragment) GN=THY1	17 kDa	0	0	**0.0222**	0
H2A2B	Histone H2A type 2-B GN=HIST2H2AB	14 kDa	0	0	**0.0262**	0
ALDR	Aldose reductase GN=AKR1B1	36 kDa	0	0	0	**0.007**
CBPM	Carboxypeptidase M GN=CPM	51 kDa	0	0	0	**0.005**
B1A4H2	Chorionic somatomammotropin hormone 1 GN=CSH1	14 kDa	0	0	0	**0.018**
FBN1	Fibrillin-1 GN=FBN1	312 kDa	0	0	0	**0.002**
E9PCY7	Heterogeneous nuclear ribonucleoprotein H GN=HNRNPH1	47 kDa	0	0	0	**0.005**
CD44	Isoform 15 of CD44 antigen GN=CD44	32 kDa	0	0	0	**0.004**
PZP	Isoform 2 of Pregnancy zone protein GN=PZP	140 kDa	0	0	0	**0.003**
ENOB	Isoform 3 of Beta-enolase GN=ENO3	42 kDa	0	0	0	**0.017**
NNRD	Isoform 4 of ATP-dependent (S)-NAD(P)H-hydrate dehydratase GN=CARKD	25 kDa	0	0	0	**0.009**
E9PH82	Protein FAM98A GN=FAM98A	34 kDa	0	0	0	**0.014**
ESTD	S-formylglutathione hydrolase GN=ESD	31 kDa	0	0	0	**0.015**
TBB3	Tubulin beta-3 chain GN=TUBB3	50 kDa	0	0	0	**0.007**
TBB8	Tubulin beta-8 chain GN=TUBB8	50 kDa	0	0	0	**0.002**
SYNC	Asparagine--tRNA ligase, cytoplasmic GN=NARS	63 kDa	0	0	0	**0.008**
IGSF3	Immunoglobulin superfamily member 3 GN=IGSF3	135 kDa	0	0	0	**0.004**
ITPA	Isoform 2 of Inosine triphosphate pyrophosphatase GN=ITPA	20 kDa	0	0	0	**0.018**
SNX1	Sorting nexin-1 GN=SNX1	59 kDa	0	0	0	**0.008**
TXNL1	Thioredoxin-like protein 1 GN=TXNL1	32 kDa	0	0	0	**0.011**
TPBG	Trophoblast glycoprotein GN=TPBG	46 kDa	0	0	0	**0.016**

Supplementary Table 3: Clathrin-mediated endocytosis related proteins identified as O-GlcNAc-modified in T1D or T2D placental lysates, following enrichment of modified proteins using sWGA. Protein abundance quantified using the normalised spectral abundance factor (NSAF). NSAF is defined as a ratio of the number of spectra identifying a protein divided by the protein length expressed as the number of amino acids.

		T1D comparison	T2D comparison	Uniquely expressed	
**Symbol**	**Protein Name**	**Fold Change**	**Fold Change**	**T1D**	**T2D**
ACTG2	actin, gamma 2, smooth muscle, enteric	4.002	ns		
ACTR2	ARP2 actin-related protein 2 homolog (yeast)	ns	-	√	
ACTR3	ARP3 actin-related protein 3 homolog (yeast)	3.13	ns		
AP1B1	adaptor related protein complex 1 beta 1 subunit	-2.386	ns		
AP2A1	adaptor related protein complex 2 alpha 1 subunit	ns	ns		
AP2A2	adaptor related protein complex 2 alpha 2 subunit	-	-9.396		√
AP2B1	adaptor related protein complex 2 beta 1 subunit	ns	ns		
APOA1	apolipoprotein A-I	-1.12	1.241		
APOB	apolipoprotein B	-	-		√
APOE	apolipoprotein E	2.087	ns		
ARF6	ADP ribosylation factor 6	2.087	-2.349		
ARPC2	actin related protein 2/3 complex subunit 2	ns	ns		
ARPC3	actin related protein 2/3 complex subunit 3	3.13	2.554		
ARPC4	actin related protein 2/3 complex subunit 4	ns	ns		
CDC42	cell division cycle 42	-	-		√
CLTC	clathrin heavy chain	ns	ns		
CLU	clusterin	4.173	-2.349		
CSNK2B	casein kinase 2 beta	2.042	-	√	
DAB2	DAB2, clathrin adaptor protein	-	-1.566		√
DNM2	dynamin 2	3.13	2.554		
HSPA8	heat shock protein family A (Hsp70) member 8	ns	-	√	
ITGA5	integrin subunit alpha 5	ns	ns		
ITGB1	integrin subunit beta 1	-1.307	ns		
ITGB3	integrin subunit beta 3	-	2.554		√
ITGB4	integrin subunit beta 4	-3.344	ns		
LYZ	lysozyme	ns	ns		
PICALM	phosphatidylinositol binding clathrin assembly protein	2.087	-	√	
RAB11A	RAB11A, member RAS oncogene family	ns	-5.481		
RAB5B	RAB5B, member RAS oncogene family	ns	-2.104		
RAB5C	RAB5C, member RAS oncogene family	ns	-4.698		
RAB7A	RAB7A, member RAS oncogene family	ns	ns		
RAC1	ras-related C3 botulinum toxin substrate 1 (rho family, small GTP binding protein Rac1)	ns	ns		
S100A8	S100 calcium binding protein A8	2.087	-	√	
TF	transferrin	ns	19.455		
TFRC	transferrin receptor	ns	ns		
USP9X	ubiquitin specific peptidase 9, X-linked	ns	ns		

*More O-GlcNAc-modified proteins (**red**) and less O-GlcNAc-modified proteins (**green**). ns - Protein abundance that did not reach significance cut off of 2-fold.* ***√*** *- Unique proteins identified by type of diabetes*

Supplementary Table 4: MASCOT search parameters used

Protein Identification and quantification	
iTRAQ discovery:	
Raw data files from the MS (.dat files) were converted to MGF format and searched, using MASCOT against the Swissprot database (2012_11) with Taxonomy set to humans.	
Fasta file:	UniProt_Human_2013_10.fasta
Fixed modifications:<br>-identifier, site, delta (neutral/loss).	Carbamidomethyl cysteine (C) +57.021464
Variable modifications:<br>-identifier, site, delta (neutral/loss(es)).	Dehydrated serine (S) -18.010565<br>Dehydrated threonine (T) -18.010565<br>Oxidation (M) +15.994915<br>Phospho serine, threonine (ST) +79.966331, 0, 97.976896<br>Phospho tyrosine (Y) +79.966331<br>HexNAc asparagine (N) +203.079373<br>HexNAc serine (S) +203.079373<br>HexNAc threonine (T) +203.079373<br>Acetyl (N-term) +42.010565
Enzyme:	Trypsin
Maximum missed cleavages:	1
Peptide mass tolerance:	10
Peptide mass tolerance Units:	ppm
Fragment mass tolerance:	0.3
Fragment mass tolerance unit:	Da
Mass Values:	Monoisotopic
Instrument type:	5600 TripleTOF mass spectrometer
Decoy database also searched:	1
Significance threshold:	0.01

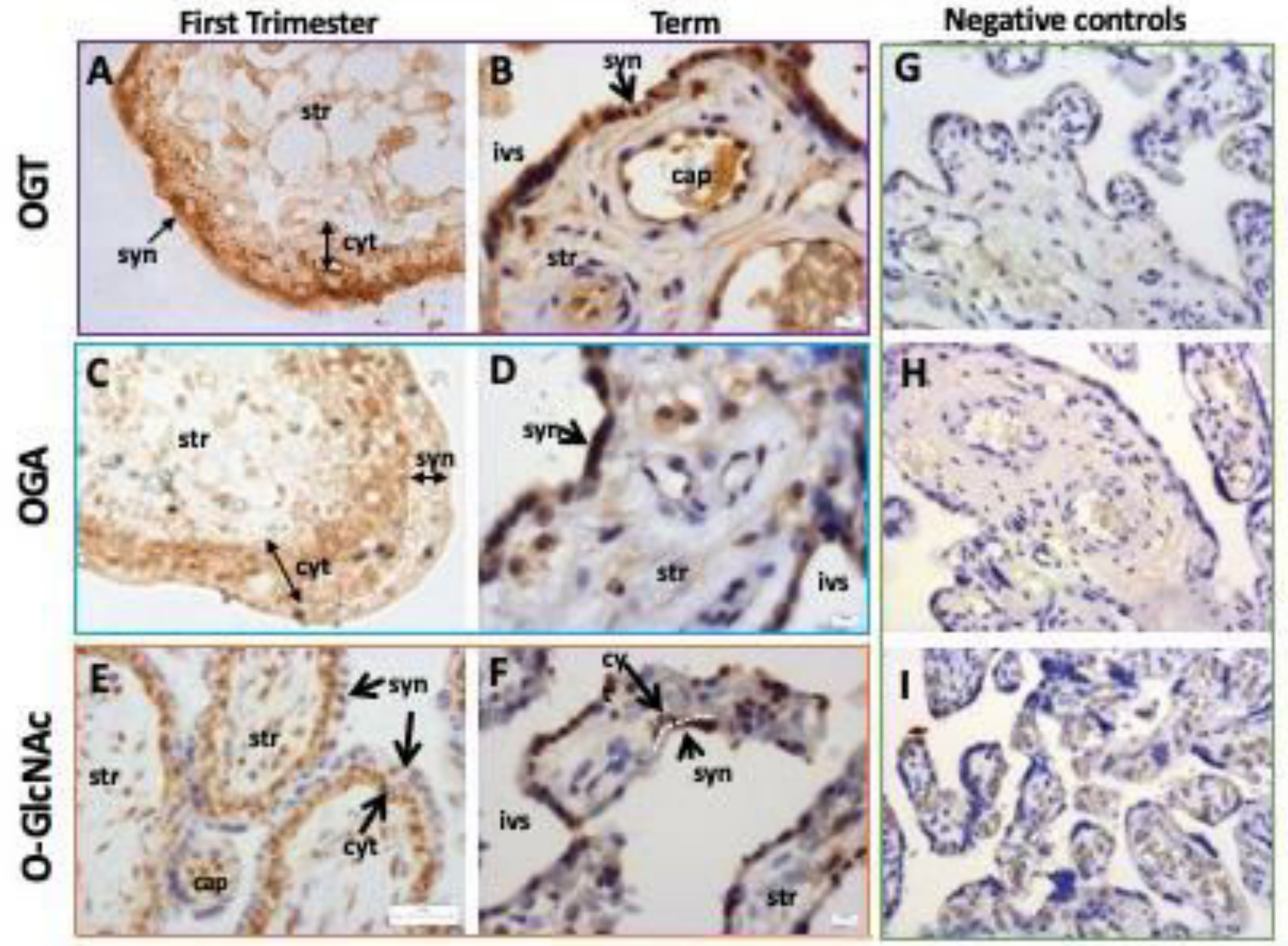

Supplementary Figure 1: Expression of O-GlcNAc modifying enzymes and O-GlcNAcylated proteins in human placenta for first trimester and term placenta. Localisation was determined by immunostaining of human placentas obtained from uncomplicated pregnancies in first trimester and at term counterstained with haematoxylin. Staining for O-GlcNAc enzymes: OGT **(A & B)**; OGA: **(C & D)**; and O-GlcNAc-modified proteins **(E & F)**. Negative controls **(G-I)** were generated by omitting the primary antibody before incubation with appropriate secondary antibody.
Abbreviations: syn – syncytiotrophoblast, cyt – cytotrophoblast, ivs – intervillous space, str – stroma, cap – fetal capillary, dashed line – syn-cyt interface. Images are representative of multiple tissues (n=4).

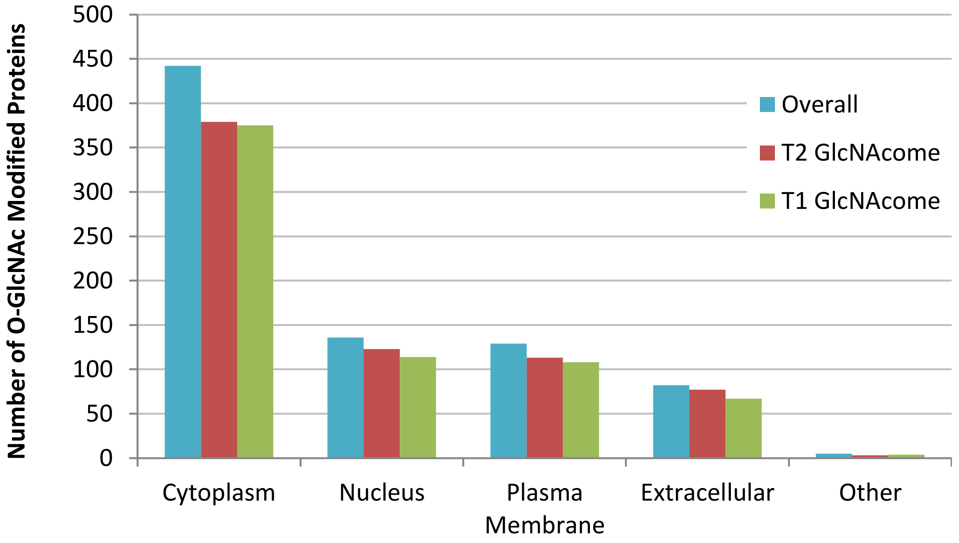

Supplementary Figure 2: The proportion of O-GlcNAc-modified proteins identified by mass spectrometry in relation to their cellular location overall and by T1D and T2D placental samples.

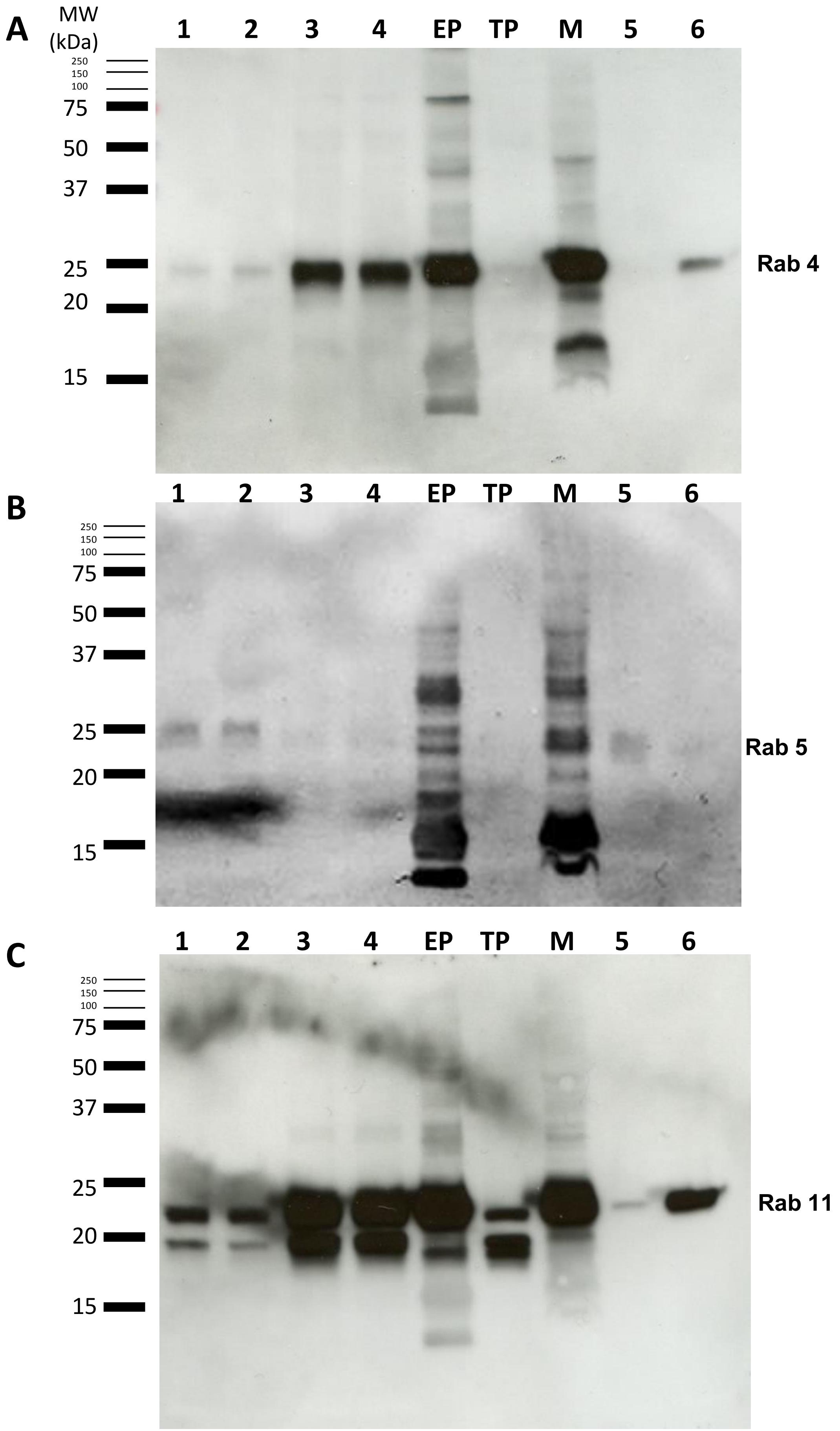
A
MW (kDa)
1 2 3 4 EP TP M 5 6
250
150
100
75
50
37
25
20
15
Rab 4
B
1 2 3 4 EP TP M 5 6
250
150
100
75
50
37
25
20
15
Rab 5
C
1 2 3 4 EP TP M 5 6
250
150
100
75
50
37
25
20
15
Rab 11

D

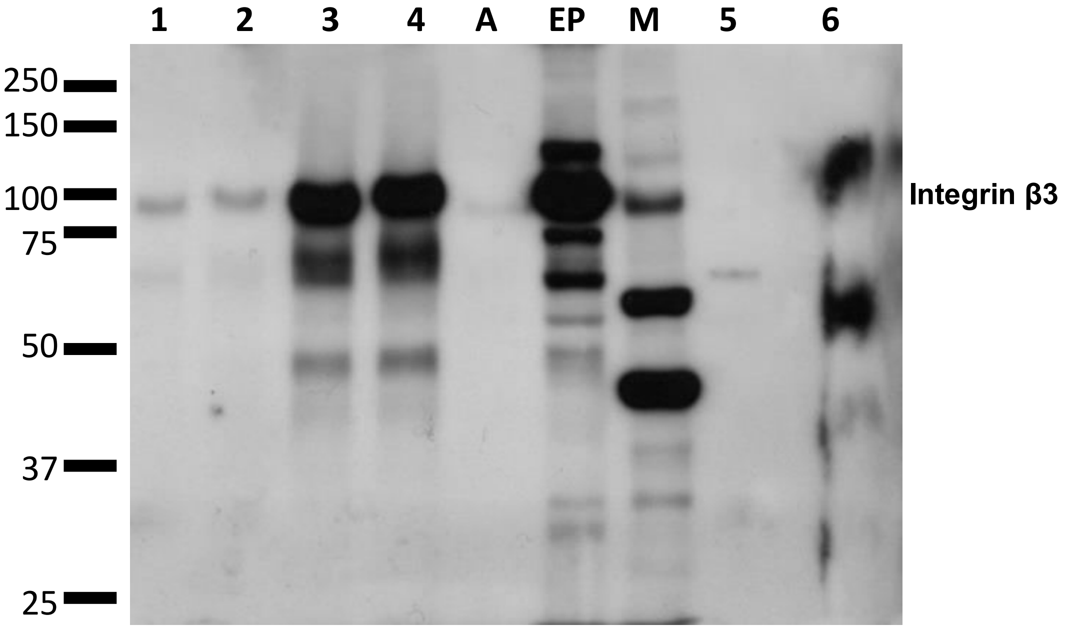
1 2 3 4 A EP M 5 6
250
150
100
75
50
37
25
Integrin β3

E

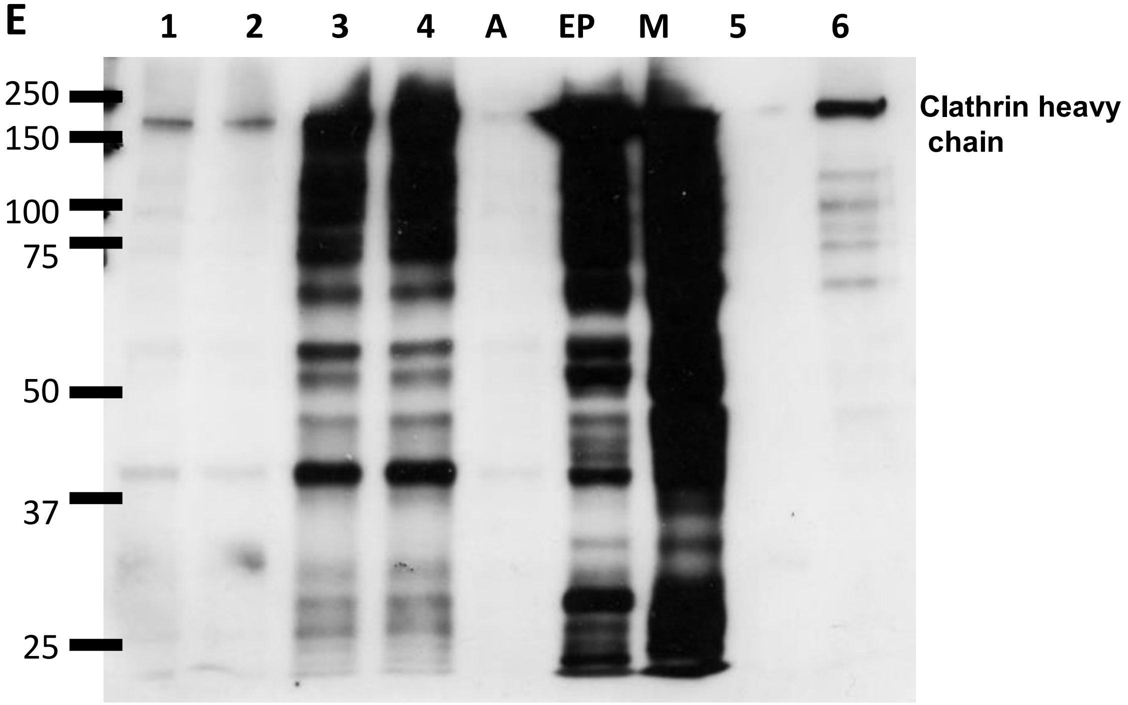
1 2 3 4 A EP M 5 6
250
150
100
75
50
37
25
Clathrin heavy chain

Supplementary Figure 3: Components of clathrin-mediated endocytosis are O-GlcNAc modified. Western blot analysis of O-GlcNAc-modified proteins isolated by sWGA-lectin pulldown from term placenta lysates (n=6; pooled) obtained from mothers with T2D (1), or BMI-matched control (2). The remaining supernatants, depleted of O-GlcNAc proteins (lanes 3 and 4, respectively). (A) Plain, unconjugated agarose beads exposed to tissue lysate and precipitate were used as a negative control, loaded to show any nonspecific binding. Three positive controls (40µg each): (EP) first trimester human placenta (TP) term human placenta and (M) mouse brain were loaded to demonstrate the specificity of the primary antibodies. Lane (5) BeWo lysate, from control untreated cells, following sWGA-lectin enrichment and (6) depleted BeWo supernatant. Membranes were probed with antibodies specific for **(A)** RAB4, **(B)** RAB5, **(C)** RAB11, **(D)** integrin β3 and **(E)** clathrin heavy chain.

A)

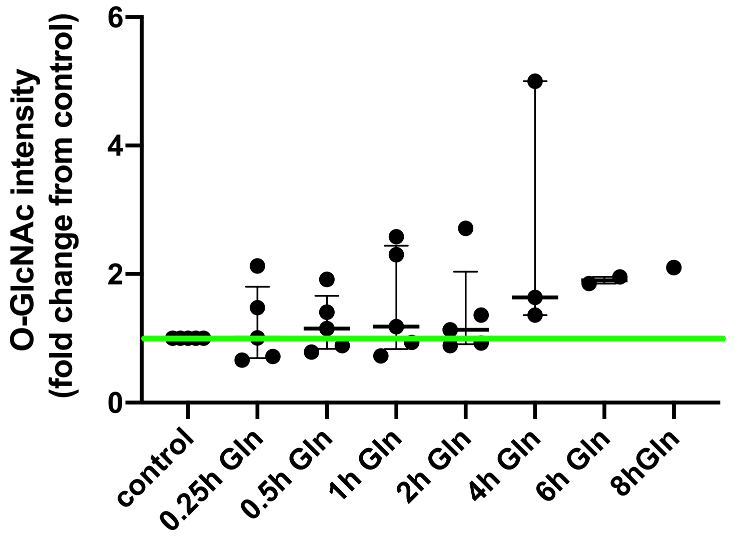

B)

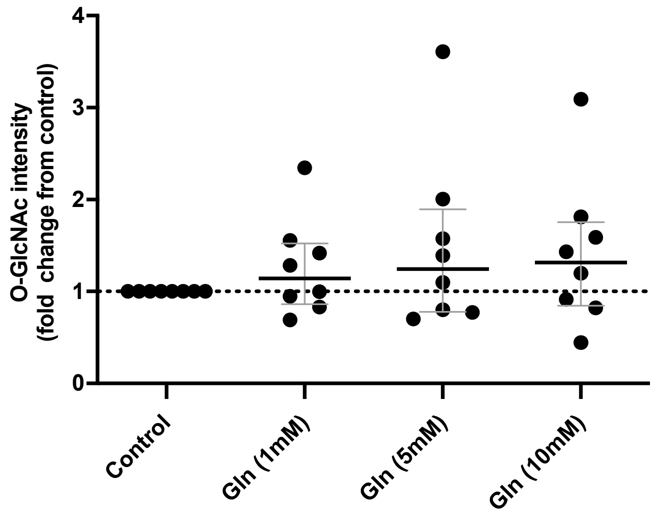

Supplementary Figure 4: Manipulating nutrient flux through the hexosamine biosynthetic pathway (HBP):

(A) BeWo cells (n=3) were exposed to glucosamine treatment (Gln; 2.5mM) for time periods ranging from 0.25-8 hours. **(B)** First trimester human placental explants (n=8) were treated with glucosamine (1-10mM) for 48h. Western blot analysis was conducted for total protein O-GlcNAcylation using a specific anti-O-GlcNAc antibody (Sigma; UK). Data are displayed as fold change in O-GlcNAc intensity, normalised to β-actin (median and interquartile range).

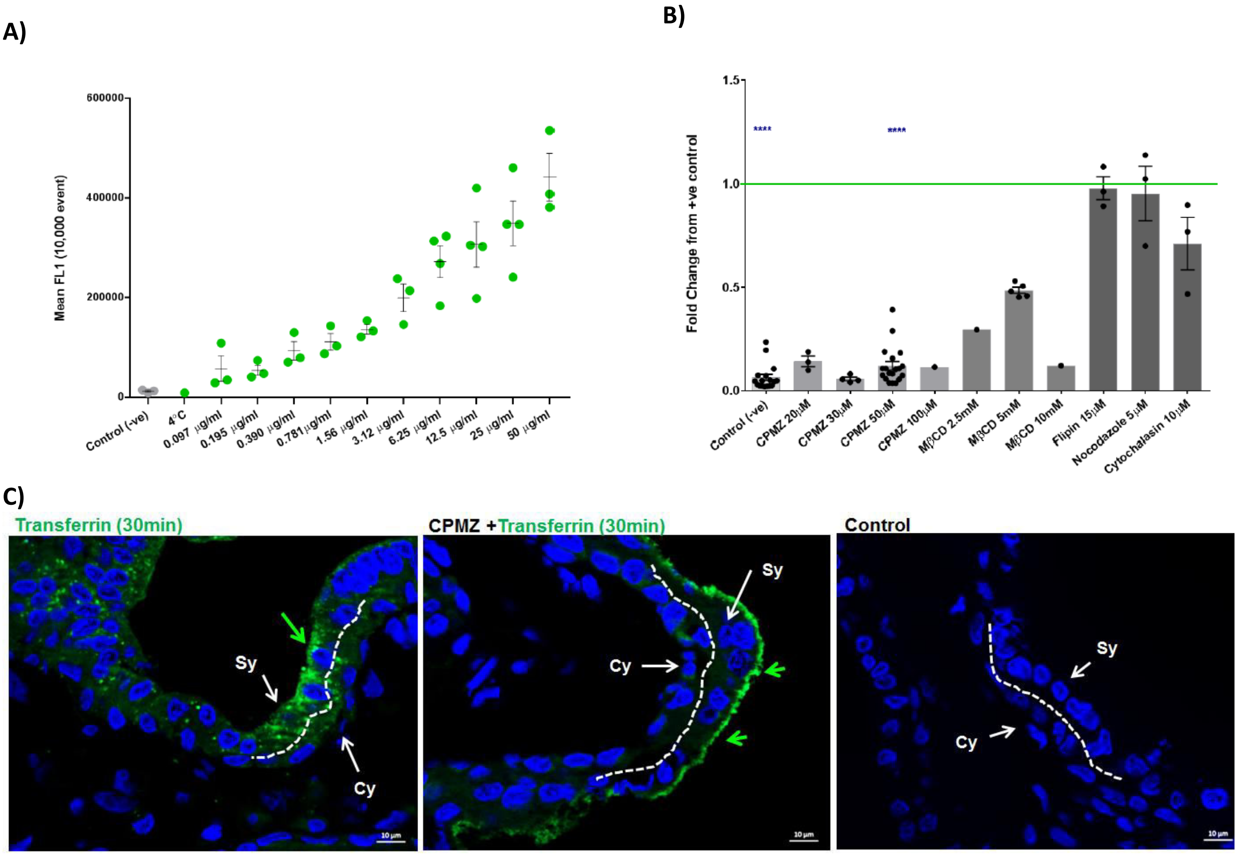

Supplementary Figure 5: Assessment of clathrin-mediated endocytosis of fluorescently labelled transferrin (Alexa 488) in placenta. **(A)** Cells were cultured in serum-depleted medium for one hour, placed on ice (~4°C) to stop all trafficking (5 mins) prior to transferrin treatment (0.097–50µg/ml) for 15 minutes at 37˚C. Cells were then washed with an acid solution (pH2.2) to remove extracellular bound transferrin, fixed and analysed by flow cytometry. The negative control represents background fluorescence of cells not exposed to transferrin. Data displayed as mean fluorescence in 10,000 events per treatment (n=3 or 4), Mean with SEM. **(B)** In some experiments, cells were pre-cultured with inhibitors of endocytosis for 1hr at 37°C before exposure to transferrin (15mins), acid washed and fixed. Data displayed as mean fluorescence in 10,000 events, presented as fold change from a positive control (transferrin uptake with no inhibition) shown with an intercepting line at 1. Negative control represents background fluorescence of cells not exposed to transferrin. Chlorpromazine (CPMZ) inhibits clathrin-mediated endocytosis, Methyl-β-cyclodextrin (MβCD) inhibits caveolin-dependent endocytosis and is a partial inhibitor of clathrin-mediated endocytosis. Filipin inhibits caveolin endocytosis specifically. Nocodazole disrupts the polymerisation of tubulin. Cytochalasin inhibits the polymerisation of actin. Data displayed as mean SEM. Statistical significance was determined using Wilcoxon signed ranked statistical analysis was used where * p=0.05, **p=0.01, ***p=<0.001 and ****p=<0.0001. Each data point represents an independent experiment. **(C)** Placental tissue was cultured with transferrin (50µg/ml; green) ± CPMZ (50µM) for 30 min then fixed, OCT embedded and sectioned (5µM), before mounting and staining nuclei with DAPI. Images are representative of two repeats, x63 magnification, where all scale bars represent 10µM. Sy – syncytium, Cy – cytotrophoblast. Green arrows highlight fluorescent transferrin.
